# Defining key questions for clinical practice guidelines: a novel approach for developing clinically relevant questions

**DOI:** 10.1186/s12961-020-00628-3

**Published:** 2020-09-29

**Authors:** Samantha Chakraborty, Bianca Brijnath, Jacinta Dermentzis, Danielle Mazza

**Affiliations:** 1 Department of General Practice, Faculty of Medicine, Nursing and Health Sciences, School of Primary and Allied Health Care, Monash University, Building 1, 270 Ferntree Gully Road, Notting Hill, Victoria 3150 Australia; 2 National Ageing Research Institute Ltd, Parkville, Australia

**Keywords:** Clinical judgement, Key clinical questions, Guideline scope, Clinical practice guideline, Implementation, General practitioner, GP, Guideline methodology, Guideline manual

## Abstract

**Background:**

There is no standardised protocol for developing clinically relevant guideline questions. We aimed to create such a protocol and to apply it to developing a new guideline.

**Methods:**

We reviewed international guideline manuals and, through consensus, combined steps for developing clinical questions to produce a best-practice protocol that incorporated qualitative research. The protocol was applied to develop clinical questions for a guideline for general practitioners.

**Results:**

A best-practice protocol incorporating qualitative research was created. Using the protocol, we developed 10 clinical questions that spanned diagnosis, management and follow-up.

**Conclusions:**

Guideline developers can apply this protocol to develop clinically relevant guideline questions.

## Background

Clinical guidelines have the potential to translate knowledge into practice through rigorous assessment of the medical literature and to help to establish norms of practice for a clinical topic [1]. General practitioners (GPs) experience challenges in diagnosing and managing mental health conditions that have arisen as a result of work and, in Australia, GPs have requested clinical guidance to assist them in providing high quality care to their patients [2]. As a result, our team was commissioned to create a clinical guideline for GPs on the diagnosis and management of mental health conditions that have arisen as a result of work; we were determined to create an implementable guideline.

Guideline implementation begins at the start of and continues throughout the guideline development process, into post-publication [3–5]. In 2012, the Guidelines International Network endeavoured to standardise guideline development processes internationally by publishing international standards for clinical practice guidelines [6]. Whilst there are numerous international protocols for developing high-quality clinical practice guidelines (e.g. those published by the National Institute for Health and Care Excellence (NICE) [7], National Health and Medical Research Council [8] and WHO [9]) that adhere to these standards, for some steps in guideline development, such as developing key clinical questions for guidelines, there is no consistent protocol [10].

In fact, following a recent review of prioritisation exercises in published guidelines [10, 11], the authors identified 11 steps of prioritisation that were used by guideline developers and noted that these steps are used inconsistently across published studies [10, 11]. This lack of a standardised process may produce guidelines that are not relevant for end-users or do not address their clinical needs. For example, when describing barriers to guideline implementation, clinicians highlight other clinical concerns that they prioritise in practice and for which guidance does not exist [12]. By incorporating clinician views into priority-setting and the development of key clinical questions in a standardised way, we may create more useful and implementable guidelines. One way to incorporate clinician views into this process is to embed qualitative research into the process of formulating key clinical questions because qualitative research can bring a real-world context to the process [13].

### Aim

Our aim, therefore, was to create a protocol to develop key clinical questions that incorporates qualitative research with end-users and to apply this method in developing key clinical questions for a new guideline.

## Methods

### Study context

In Australia, GPs who see patients with work-related mental health conditions have asked for guidance on how to effectively diagnose and manage work-related mental health conditions [2]. In response to this call to action, our team commenced developing national clinical guidelines for GPs in accordance with the Australian gold standard approach for guideline development outlined by the Australian National Health and Medical Research Council [8]. We considered it critical that the guideline addressed the clinical needs of the intended end-users of this guideline — GPs.

### Qualitative approach and research paradigm

To develop the protocol, we first undertook a scoping review of published guideline development manuals to identify best-practice approaches for guideline development and combined these to construct a foundation protocol. We then augmented the foundation protocol with qualitative research to identify and incorporate clinical challenges into the process. Finally, we tested the feasibility of the protocol by using it when creating key clinical questions for the new clinical guideline. Before we elaborate on these steps in detail below, it is important to note that there is no established method for developing protocols to enhance guideline development; however, our methods align with existing guidance for developing clinical checklists [14, 15]. We use the Standards for Reporting Qualitative Research to describe this research [16].

### Step 1: develop a foundation protocol based on best practice guideline development manuals

#### Data collection methods

In April 2016, we undertook a scoping search of major guideline enterprises in Europe, the United Kingdom, the United States and Australia to identify published guideline development manuals. First, we reviewed guideline databases, including the Guideline International Network and the National Guideline Clearinghouse, and supplemented this with a Google search to identify major guideline enterprises. Guideline enterprises were defined as organisations who oversee guideline development or the approval of clinical guidelines. Subsequently, we reviewed the websites of these organisations and supplemented with a Google search to identify published guideline development manuals from these organisations. We used an iterative process that commenced with two members of the team (DM and SC) compiling an initial list of key guideline enterprises and searching the websites of these organisations to identify the most recent version of their guideline development manuals. In addition, we undertook a scoping search on Medline to identify other published guideline development manuals or reports [17, 18]. Manuals that described protocols for developing key clinical questions were included.

#### Data processing and analysis

To develop the foundation protocol, two members of the team (SC and JC) reviewed the identified guideline development manuals and extracted from them instructions for developing key clinical questions. All authors then discussed the extracted instructions and selected the most comprehensive protocols to form a foundation protocol.

### Step 2: augment the foundation protocol with qualitative research to identify and incorporate clinical challenges into the process

Thereafter, we reviewed the foundation protocol and considered where, in that protocol, a qualitative study would be most useful and what qualitative design would assist in identifying clinical challenges into the process. A decision was made to conduct qualitative interviews with GPs, who were the targeted end-users of the guideline, as well as with other key informants who are familiar with the challenges faced by GPs, including workers’ compensation scheme representatives and independent medical examiners, who frequently liaise with GPs to assess claims and facilitate return to work for patients [19]. We provided interviewees with two previously validated case studies that described common patient journeys from their first consultation with a GP until 12 months later [20] and we asked interviewees to reflect on the clinical dilemmas that GPs face in practice when dealing with these conditions. To facilitate this reflection, we asked GPs to discuss where throughout the clinical consultation they would refer to a clinical guideline, if one existed. Conversely, we asked compensation scheme representatives and independent medical examiners to discuss what they perceived as shortcomings in clinical practice, using the scenarios provided [19].

Together with our qualitative findings gleaned from this process [19] to augment the foundation protocol, we analysed these data using a clinical reasoning framework. By using clinical reasoning as a thematic framework to categorise these challenges, we were able to arrange these challenges according to the practical stages of a clinical consultation. This layout was applied to the presentation of topics in the guideline to create a document that aligns with the progression of clinical dilemmas that GPs are likely to face during consultations with patients. In addition to applying this framework, there was discussion and feedback between the authors (including DM, who is a practicing GP). We reviewed the foundation protocol to identify the steps in this protocol that could reveal the clinical concerns faced by potential clinician end-users. We then modified the identified steps to incorporate qualitative research, particularly clinical reasoning, at these steps. This method allowed us to separate clinical issues from systemic ones so that the clinical dilemmas could be addressed in the guideline.

### Case study

We applied the augmented protocol to develop key clinical questions for this new clinical practice guideline. The guideline project team was responsible for applying the protocol to develop key clinical questions – this included outlining the steps for the guideline development group and providing draft responses to the steps outlined in the protocol. The guideline project team constituted two project leads (DM and BB), the project manager (SC) and the project officer (JD). The guideline development group was subsequently responsible for complying with the protocol, reviewing draft responses, revising responses and deciding on the final list of key clinical questions. The guideline development group was chaired by DM and its members included BB, SC, two additional GPs, a psychiatrist, a clinical psychologist, an occupational physician, a consumer with a lived experience of a work-related mental health condition and two policy representatives (representing Commonwealth and state-based compensation schemes in Australia).

## Results

### Step 1: develop a foundation protocol based on best practice guideline development manuals

Through the scoping search, we identified 10 guideline development manuals that described methods for developing key clinical questions (Additional File 1). Various degrees of detail were provided in these guideline manuals. Most manuals [3, 4, 8, 21–23] provided criteria to assist developers to determine whether a topic should be addressed, while others [7, 9, 24, 25] provided detailed steps for formulating key clinical questions. The content and structure of frameworks across the guideline development manuals were largely consistent and promoted three main approaches, namely (1) use a consensus-based approach to formulate key clinical questions [4, 22, 25–27]; (2) where possible, enhance the consensus-based approach with research evidence [7, 9, 21, 28]; and (3) enable the guideline development group to consider topics that are suggested by members or other informants [7, 9, 21, 25, 27].

The WHO and the NICE guideline development manuals had the most comprehensive descriptions for developing key clinical questions (Additional File 1). The WHO manual describes a seven-step method, whereas the NICE manual describes a four-step method that has a stronger emphasis on stakeholder consultation. The WHO and NICE methods for developing key clinical questions were combined into a foundation protocol combining Step 1 from the NICE manual and Steps 2–7 from the WHO manual.

### Step 2: augment the foundation protocol, drawing on a clinical reasoning framework

The foundation protocol (henceforth referred to as ‘the protocol’) comprises seven steps and includes a qualitative study using clinical reasoning at step 2 (Table 1). The seven steps of the protocol are as follows:

- *Step 1*. State the rationale for the guideline – informed by the remit of the guideline developers, undertake a detailed needs analysis of patient outcomes, clinical practice, relevant policy and other research evidence to identify the patient, clinical and policy needs that the guideline should address. A needs analysis may include a scoping search of the literature to identify key clinical issues in the care pathway, relevant clinical guidelines, health technology assessment reports, relevant systematic reviews and economic evaluations [7].
- *Step 2.* Generate an initial list of questions based on clinical challenges expressed by stakeholders. This step involves three sub-steps, as follows: (1) Undertake a qualitative research study using clinical scenarios (case vignettes) to elicit reflections on clinical care from clinicians and other relevant stakeholders, and analyse reflections using an inductive thematic approach to identify clinical challenges. (2) Review published literature, existing guidelines and policies to identify additional research evidence about clinical challenges as well as existing advice. (3) Map the clinical challenges raised during the qualitative study and existing literature against a clinical reasoning framework. These clinical challenges were mapped against a clinical reasoning framework that is described in a core general practice textbook, *Murtagh’s General Practice* 6th Edition [29] (Box 1). This clinical reasoning framework involves two key phases of the consultation – establishing rapport and diagnosis and the management phase. The diagnostic phase involves taking the patient’s history, undertaking the physical and mental examination, and conducting investigations. The management phase includes explaining the diagnosis to the patient, providing education to the patient about the diagnosis, prescribing medication, conducting procedural activities, referring patients to members of the care team and monitoring progress in a patient’s condition.
- *Step 3*. Convert the initial list of clinical questions into a PICO (Population, Intervention, Comparator, Outcome) format.
- *Step 4.* Specify all relevant outcomes for each possible question.
- *Step 5*. Revise the clinical questions in light of the outcomes identified in Step 4.
- *Step 6.* Rate the outcomes across all questions in order of importance for clinical decision-making.
- *Step 7.* Decide on the final set of key clinical questions that the guideline will address.

**Table 1:** Protocol for developing clinically relevant key clinical questions

Step heading	Description
Step 1 Define the rationale for the guideline	Undertake a detailed needs analysis of patient outcomes, clinical practice, relevant policy and other research evidence to explain the need for a guideline
Step 2 Use qualitative research methods to determine the initial list of key questions based on the clinical challenges faced by target end-users	Present clinical scenarios (case vignettes) to target end-users and other key stakeholders Utilise an inductive thematic analysis approach to identify areas of clinical concern Map areas of clinical concern against a Clinical Reasoning Framework Generate an initial list of questions based on results from the qualitative study and extend these findings with research evidence about existing clinical challenges
Step 3 Convert the initial list of questions	Convert the initial list of questions developed in Step 2 into a Population, Intervention, Comparator, Outcome (PICO) format
Step 4 Specify all relevant outcomes for each possible question	This includes not only those specified in PICO but other positive and negative outcomes
Step 5 Review and revise draft key questions	Review and revise draft key questions, in light of the specific outcomes identified in step 4
Step 6 Rate the outcomes in order of importance for clinical decision-making	As part of this step, the Guideline Development Group may consider whether advice (in the form of high-quality clinical guidelines) already exists to answer the clinical challenges that were revealed during the qualitative study
Step 7 Decide on the final list of questions	Final questions are to be decided upon based on resource availability, which can be difficult to anticipate; specifically, the resource requirements for undertaking systematic literature reviews

### Pilot testing the new protocol – a case study

We developed key clinical questions using the protocol over a period of 6 months (Additional File 2). First (Step 1) a scoping search of the literature identified broad challenges with assessment, diagnosis, management and follow-up related to work-related mental health conditions, with an emphasis on acute stress disorder, post-traumatic stress disorder, anxiety, depression, adjustment disorder and substance misuse. Then (Step 2) qualitative interviews with GPs, workers’ compensation scheme representatives and independent medical examiners assisted in identifying a total of 19 clinical challenges [19] and an additional 2 challenges were identified through a search of published literature. Thus, 21 clinical challenges were associated with diagnosing and managing work-related mental health conditions in general practice. When mapped onto the clinical reasoning pathway, these challenges represented all stages of the pathway. In addition to the clinical challenges, interviewees also described a number of system complexities that interact with the GP’s ability to provide high quality clinical care to patients, for example, uncertainty about roles and responsibilities. Such issues did not fit neatly into a single step of the clinical reasoning pathway but were applicable across the pathway. Having clearly defined the clinical challenges, the project team then transformed the clinical concerns into initial questions without difficulty.

**Fig. 1:**
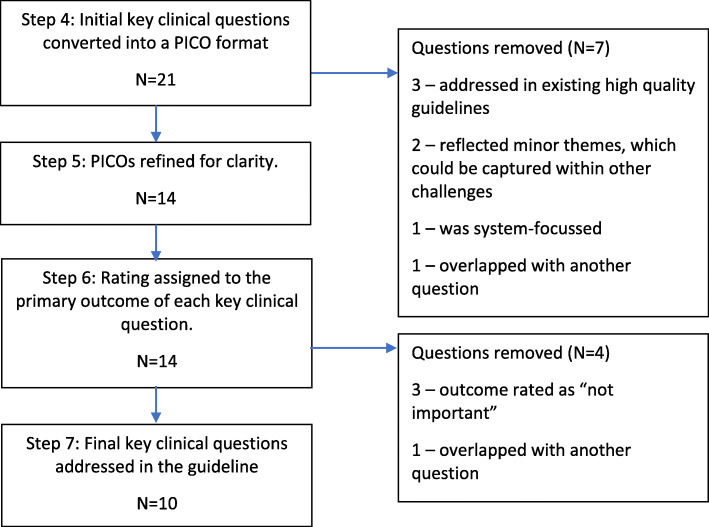
Refining key clinical questions from an initial list of clinical challenges (*N* number of questions)

Next, the project team converted the initial questions into a PICO format (Step 3). In the absence of a published core outcome set for the topic of work-related mental health conditions, we reflected on outcomes described in published research on the topic [2, 19, 30, 31] to develop an initial list of relevant outcomes for each possible question (Step 4). A total of 21 questions were created. The results of steps 1 to 4 were presented to the guideline development group at a face-to-face meeting, at which point the guideline development group reviewed the initial list of key clinical questions and then completed steps 5 to 7 (Fig. 1). The guideline development group removed seven initial questions because they were addressed in existing high-quality clinical guidelines (three questions), reflected minor themes (two questions), were system-focused and therefore considered as not appropriate for a clinical guideline (one question), or overlapped with another question (one question). In step 5, following discussion and revision in steps 1–4, the guideline development group edited the wording of the remaining 14 initial clinical questions. In step 6, the guideline development group assigned a rating to the primary outcomes (where 1 was considered as ‘not important’ and 5 was considered as ‘very important’) for these 14 questions. The guideline development group scored the importance of primary outcomes as a group and reached consensus about the rating of each outcome in turn before moving to the next. However, our group scored most primary outcomes as either ‘very important’ or ‘not important’, with only one topic receiving another rating. Questions with a rating of ‘not important’ (three questions), or where there was overlap, were removed. In the final step, the group chose to include all questions with high-rated outcomes. A total of 10 key clinical questions were selected, as follows:

1. In workers presenting with symptoms of mental health conditions, what tools can assist a GP to make an accurate (sensitive and specific) diagnosis of a mental health disorder and its severity?
2. In workers, what factors assist in the early detection of a comorbid work-related mental health condition?
3. In patients with a diagnosed mental health condition, what methods are effective at indicating the probability that the diagnosed mental health condition has arisen as a result of work?
4. When conveying a diagnosis of a work-related mental health condition to a patient, what factors should GPs consider, to ensure that their diagnosis is understood and acknowledged by the patient?
5. In patients with a work-related mental health condition, what GP strategies result in the highest level of personal recovery and/or return to work?
6. In workers with a mental health condition, what information should a GP consider to determine whether a person has the capacity to work?
7. What is appropriate communication with the patient’s workplace in order to appropriately manage a work-related mental health condition?
8. In patients with a work-related mental health condition, what GP interventions are effective at managing comorbid substance misuse and addictive disorders?
9. In patients with a diagnosis of a work-related mental health condition, what factors adversely affect progress in the patient’s condition?
10. In patients with work-related mental health conditions who are not improving, what strategies should a GP undertake to improve the patient’s condition?

Box 1: Clinical reasoning framework for use by general practitioners when consulting with all patients. The components of the framework are based on Murtagh’s General Practice [29] Diagnosis:

 1) Thorough but directed clinical history, with initial hypothesis generation and subsequent testing

  a) What is the probability diagnosis?

  b) What serious disorders must not be missed? (What are the ‘red flags’?)

  c) What conditions are often missed (the pitfalls)?

  d) Could this patient have one of the ‘masquerades’ in medical practice?

  e) Is this patient trying to tell me something else?

 2) Primary diagnosis and differential diagnosis in order of likelihood

  a) Look for symptoms and risk factors to decide if a diagnosis should be still considered

 3) Physical examination to get further data to confirm or refute the hypotheses

  a) Do investigations to include and exclude differential diagnoses

 4) Thoughtful and critical selection of investigations to gather additional data

  a) Systematically investigate the probability of a mental health condition

  b) Systematically investigate that the mental health condition is a result of work

Management

 5) Implementation of a targeted and rationalised management plan

  a) Tell the patient the diagnosis

  b) Establish the patient’s knowledge of the diagnosis

  c) Establish the patient’s attitudes to the diagnosis and management

  d) Educate the patient about the diagnosis

 6) A management plan for the presenting problem

   a) Consider management in the:

    i) Immediate term

    ii) Intermediate term (safety-netting: what to look for that might prompt them to come back/further review)

    iii) Long-term

    iv) Preventive

   b) Explore other preventive opportunities (e.g. addressing risk factors such as illicit drugs/alcohol)

   c) Reinforce the information

   d) Provide take-away information

   e) Evaluate the consultation (e.g. have you got any other concerns?)

 7) Arrange follow-up

Important consideration: weighting of evidence provided by a patient

## Discussion

In this study, we developed a protocol for creating key clinical questions by combining best-practice approaches and augmenting the combined approach with a clinically relevant qualitative research study. We then successfully used the protocol to develop the key clinical questions for a new guideline for general practitioners on the diagnosis and management of work-related mental health conditions. This protocol fills the existing gap in guideline development literature to describe a best-practice approach for developing clinically relevant key clinical questions for guidelines. Until now, guideline developers have used varying methods to develop key clinical questions for guidelines. A best-practice approach combines high quality guideline manuals to offer a single method that can be applied and evaluated by guideline developers and researchers [18].

The resulting protocol for developing key clinical questions for guidelines is valid because it takes a step-wise approach based on best practice and qualitative research methods that are relevant to practicing clinicians. While there is no established method for developing key clinical questions for guidelines, this protocol draws on two guideline development manuals that are highly regarded internationally (i.e. manuals used by WHO [9] and NICE [7]). There is no international criteria to assess the quality or usability of instructions for developing guidelines [32, 33]; therefore, the determination of best-practice had to be a subjective assessment. To prevent any biased views in selecting the best-practice manuals, we chose to search for manuals published by large guideline enterprises and we then selected the manuals with the most comprehensive instructions for developing key clinical questions – the WHO and NICE manuals – for the basis of the protocol. We can therefore be confident that the protocol reflects best practice.

In addition to using current best practice approaches as its foundation, the protocol also incorporates a rigorous qualitative research element that comprises primary and secondary sources of evidence (interviews with end-users and stakeholders and existing literature and guidance, respectively) and, importantly, a clinical reasoning framework. Previously, guideline developers have used either secondary sources of qualitative evidence and/or primary sources of qualitative research evidence to develop key clinical questions [13, 28, 34]. Secondary sources, such as literature reviews and existing guidelines, can reveal key clinical concerns that have been documented and sometimes addressed through guidance; however, these do not take into consideration the pressing clinical concerns faced by potential end-users of a guideline. Initiatives such as the Grading of Recommendations Assessment, Development and Evaluation (GRADE) working group [35] and others (e.g. the WHO-INTEGRATE (INTEGRATe Evidence) [36, 37]) have created Evidence to Decision Frameworks (EtDs) that can be applied at the guideline scoping stage to frame clinical questions so that they are useful to end-users. Whilst EtDs are now widely used in guideline development, they are often described in the context of developing recommendations, rather than developing clinical questions. The protocol described here complement EtD, as it brings to the forefront issues that are likely to be considered in EtDs during the later recommendation development stage.

Guideline best practice involves meaningful stakeholder use throughout development [3]. For instance, stakeholder involvement in guideline development increases agreement with the guideline recommendations [38] and the ability to overcome the inertia of previous practice [39]. The use of published core outcome sets, which are developed through rigorous stakeholder engagement, are particularly useful when defining the outcomes of a PICO and are now regular practice of some guideline enterprises (e.g. NICE [7]). However, where such core outcome sets do not exist or do not capture the views of all relevant stakeholders, a qualitative research element can provide a useful and rigorous element to the development of outcomes that are relevant for end-users.

By undertaking interviews with the target end-users of the guideline and other stakeholders and by using a coding framework that is analogous with a clinician’s practice, guideline developers can identify key clinical concerns that are relevant to their target end-users. The clinical reasoning framework that we used may be relevant for other clinician groups. The principles of clinical reasoning – collecting information, developing a hypothesis, testing the hypothesis, reanalysis, making a differential diagnosis, deciding on a treatment approach and reviewing patient outcomes – are consistent across specialities [40]. The framework used in our pilot study would therefore be useful in the development of guidelines for multiple clinical specialities.

The protocol described here can be used to develop clinically relevant key clinical questions. Through our application of this protocol, our team was able to obtain a granular understanding of the clinical challenges that were hampering care in practice, where this was not initially evident in existing research. For example, our scoping study revealed that GPs and other key stakeholders identified challenges with the diagnosis and management of work-related mental health conditions in general practice, but there was little clarity about the exact nature of these challenges [2]. By conducting the qualitative study within the protocol for developing key clinical questions, we were able to distil the specific challenges encountered by GPs during the provision of care for patients. We identified three points of the diagnosis that were specifically challenging in practice.

The project team and guideline development group also considered the protocol for developing key clinical questions to be feasible in practice. The steps were clearly articulated and could be undertaken with the resources and expertise of the project team and guideline development group. One potential adjustment would be to improve instructions for determining the feasibility of answering the key clinical questions (i.e. steps 6 and 7). In step 6, the team considers that it would be beneficial to not only rate the outcomes but also to rank the questions in order of importance. Thus, if outcomes for clinical questions receive a uniform rating (e.g. all considered ‘very important’) then a ranking system can highlight questions that may be excluded from the guideline. Additional advice in step 7, to determine the feasibility of conducting systematic reviews for all the identified questions, would also be useful. Morgan et al. [41] offer reasonable advice to select the final list of questions. They suggest that guideline developers undertake a scoping or realist review of the key clinical questions to determine the feasibility of answering each identified question, narrow the PICO of key clinical question that produce voluminous search findings, and seek advice from methodologists, evidence reviewers and the approving organisation to assess guideline requirements and feasibility. If a guideline development group considers that a topic is important, but a systematic literature review is not feasible, the group should consider the possibility of adopting or adapting other high-quality guidance [42]. If none of these options are possible, then developers should highlight this as an unaddressed topic in the guideline and consider the de novo development of a recommendation at a later stage.

### Strengths and limitations

A strength of the protocol is that it reveals clinical challenges that are relevant to the target end-users. Guideline enterprises consider the clinical need of a guideline in their decision about whether to develop or update a guideline for a particular topic. However, at this stage, the assessment of clinical need is high level and not specific enough to address the particular clinical challenges faced in practice. A limitation of the study is that, whilst this protocol points the focus towards feasibility, implementation, applicability and quality, these aspects were not systematically evaluated in the current study. In fact, to test whether this approach produced a more implementable guideline, we would have had to develop two guidelines, one using this approach, and another using questions that were developed using a different approach – this was simply not feasible in our context of creating a de novo guideline, but may be possible when updating guidelines. We highlight this as a potential gap and hope that guideline developers will consider evaluating the protocol to answer questions such as ‘Does this create a useful guideline?’, ‘Does this improve implementation?’ and ‘Is the protocol feasible (e.g. consideration of the expertise, cost, and time required to apply this protocol)?

We urge guideline developers to use this protocol to develop the key clinical questions for their guidelines and we urge guideline enterprises to incorporate this protocol into their guideline development manuals. However, we also advise that guideline developers do not follow the protocol rigidly but tailor the individual steps so that they complete the seven steps feasibly. For example, where a guideline is being developed because of new evidence and where clinical practices are well described in the literature and in clinical auditing systems, a comprehensive qualitative study with a range of end-users may not be necessary. Similarly, where a rapid guideline is being developed to address an urgent safety concern, developers may consider using published literature to highlight clinical issues to address. Secondly, where guideline resources are limited, guideline developers may consider conducting a smaller scale qualitative study that is more feasible.

## Conclusions

Until now, there was no established protocol for developing key clinical questions for guidelines. The protocol described here is built on best practice and has been piloted successfully to create key clinical questions for a new guideline for GPs, thus demonstrating that it is rigorous and feasible. Future research and reflection will demonstrate its acceptability for use in the development of clinical guidelines and whether it leads to improved guideline implementation.

## Supplementary information

**Additional file 1.** Guideline development manuals and procedures that describe methods for developing key clinical questions for guidelines.

**Additional file 2.** A summary of the steps and decisions involved in developing key clinical questions for the ‘Clinical guidelines for the diagnosis and management of work-related mental health conditions’.

## Data Availability

The dataset(s) supporting the conclusions of this article are included within the article (Additional Files 1 and 2). All original data reviewed in this study are available on open-access databases (Google and/or PubMed).

## Abbreviations

EtD: Evidence to Decision framework
GP: General Practitioner
NICE: National Institute of Health and Clinical Excellence
PICO: Population, Intervention, Comparator, Outcome
