# Supplementary material for: Defining key questions for clinical practice guidelines: a novel approach for developing clinically relevant questions
Source: Health Res Policy Syst. 2020 Sep 29;18:113. doi: 10.1186/s12961-020-00628-3 (PMC7523054; doi:10.1186/s12961-020-00628-3)
Supplement: Supplementary file 1 — Additional file 1. Guideline development manuals and procedures that describe methods for developing key clinical questions for guidelines. [file 12961_2020_628_MOESM1_ESM.docx]

Additional File 1 Guideline development manuals and procedures that describe methods for developing key clinical questions for guidelines.

| - **Guideline manual producer** | - **Year published** | - **Overview of advice provided for developing guideline key clinical questions** |
| --- | --- | --- |
| American Academy of Otolaryngology—Head and Neck Surgery Foundation (AAO-HNSF)[23] | 2012 | Step 1. Guideline development group defines the scope, preliminary topics and search criteria.  Step 2. Undertake a literature search to assess the quantity and scope of randomized controlled trials to support guideline development.  Step 3. The guideline development group refines the scope in consideration of:   - Promoting appropriate care - Reducing inappropriate or harmful care - Reducing regional variations in delivery of care - Improving access to care - Educating and empowering clinicians and patients - Facilitating coordination and continuity of care - Facilitating ethical care   A preliminary topic list is developed using the PICO or PICOTS (to include **ti**me frame and **s**etting) The topics are ranked.  There may be additional consultation with peer-reviewers, board of directors, board of governors, the general public, and relevant consumer or advocacy groups. |
| American College of Cardiology Foundation and American Heart Association, Inc. (ACC/AHA)[21] | 2010 | A checklist comprising two key themes: Questions related to the guideline overall and questions related to the guideline’s clinical objectives. Specific questions related to the guideline’s clinical objectives include:  What are the important clinical objectives related to the guideline topic?  What subtopics and related topics must be included in the guideline? Are the subtopics and related topics already covered by another organization? What comorbidities are being covered or should be covered by the topic area/guideline?  Are flow diagrams appropriate to these subtopics and related topics?  What are the potential benefits and risks for individual patients associated with an intervention or procedure?  What amount of clinical flexibility is appropriate for the topic area?  What clinical options are available?  What topics have already been covered in existing ACC/AHA Guidelines? |
| American College of Physicians (ACP)[3] | 2010 | A high-level description is available as follows: Choosing a topic for a clinical practice guideline is the first step in the guideline development process. Candidate topics come from surveys of ACP members, other clinicians, the Clinical Guidelines Committee members, and other committees and governance of the ACP. In selecting a topic, the Clinical Guidelines Committee considers the following criteria: effect of the condition on morbidity and mortality, prevalence of the condition, whether effective health care is available, areas of uncertainty and evidence that current performance does not meet best practices, cost of the condition, relevance to internal medicine, and the likelihood that evidence is available to develop recommendations. |
| Institute of Medicine [2] | 2011 | Guideline topics are prioritised using seven criteria.   1. Disease burden. Extent of disability, morbidity, or mortality imposed by a condition, including effects on patients, families, communities, and society overall. 2. Controversy. Controversy or uncertainty around the topic and supporting data. 3. Cost. Economic cost associated with the condition, procedure, treatment, or technology related to the number of people needing care, unit cost of care, or indirect costs. 4. New evidence. New evidence with the potential to change conclusions from prior assessments. 5. Potential impact. Potential to improve health outcomes and quality of life; improve decision making for patient or provider 6. Public or provider interest. Consumers, patients, clinicians, payers, and others want an assessment to inform decision making. 7. Variations in care. Potential to reduce unexplained variations in prevention, diagnosis, or treatment; the current use is outside the parameters of clinical evidence.   No specific instruction for developing key clinical questions. |
| Kaiser Permanente [24] | 2012 | A high-level description is available as follows: “Once a topic has been identified, a GDT is assembled; this team helps specify the scope of the guideline, including target populations, comparative interventions, important outcomes, and other clinical issues. This process provides direction for framing specific clinical questions, which commonly address issues of risk, diagnosis, prognosis, therapy, and harm.” |
| National Health and Medical Research Council (NHMRC) Procedures and requirements for meeting the 2011 NHMRC standard for clinical practice guideline [7] | 2011 | High-level guidance is provided as follows: To be focused on health and related outcomes guidelines will:  1. Be developed around explicitly defined clinical or public health questions  2. Address outcomes that are relevant to the guideline’s expected end users  3. Clearly define the outcomes considered to be important to the person/s who will be affected by the decision, and prioritise these outcomes. |
| National Institute for Health and Care Excellence (NICE) [6] | 2014 | The scope and questions are developed over seven stages, which are described in detail.  **Stage 1: the scoping search:** A scoping search is undertaken by an information specialist and through a scoping workshop.  **Stage 2: understanding the context:** The clinical, policy and societal context is described in detail.  **Stage 3: identifying the population and key issues:** Focus on identifying areas in which providers and commissioners of care or services most need advice. A broad review process is suggested to ensure that a range of care or services are considered.  **Stage 4: planning the NICE Pathway:** Review published guidance and guidance in development to understand how the guideline recommendations are likely to relate to existing recommendations in other guidance and where they may fit into [NICE Pathways](http://pathways.nice.org.uk/).  **Stage 5: checking the population and selected key issues with stakeholders:** Seek the views of stakeholders to confirm that the population group(s) and key issues identified by the Developer are relevant and appropriate. This may include inviting registered stakeholders to a scoping workshop.  **Stage 6: consulting on the draft scope: The draft scope is made available** on the NICE website for a 4‑week consultation, and registered stakeholders and respondents are notified. The purpose of these prompts is to seek their views on key issues (such as whether the identified outcome measures are complementary to locally defined measures) and to ask what should be included or excluded. NICE asks stakeholders to suggest areas where cost savings could be achieved.  **Stage 7: finalising the scope after consultation:** The final scope is signed off by a senior member of NICE staff with responsibility for quality assurance. Once the final scope has been published no changes should be made to it except in exceptional circumstances.  It is important that the total number of questions is manageable; can be covered in the time and with the resources available; provides sufficient focus for the guideline, and covers all areas outlined in the scope. |
| Scottish Intercollegiate Guidelines Network (SIGN) [13] | 2015 | Guideline topics selected for inclusion in the SIGN programme are chosen on the basis of the burden of disease, the existence of variation in practice, and the potential to improve outcome. The following criteria are considered by SIGN in selecting and prioritising topics for guideline development:   - Areas of clinical uncertainty as evidenced by wide variation in practice or outcomes. - Conditions where effective treatment is proven and where mortality or morbidity can be reduced. - Iatrogenic diseases or interventions carrying significant risks. - Clinical priority areas for the National Health Service Scotland. - The perceived need for the guideline, as indicated by a network of relevant stakeholders.   The Guideline Program Advisory Group makes an assessment of the extent of evidence on which to base the guideline and considers whether the benefits that were likely to accrue from successful implementation of the guideline recommendations would outweigh the efforts required to develop it. |
| US Preventive Services Task Force Procedure Manual, 2015 [22] | 2015 | Detailed description of the activities undertaken by a Topic Prioritization Workgroup are described. This includes first determining if a topic is of interest and importance. Then an active prioritisation process includes the following: 1) a Background paper of the issue; 2) Review by the Topic Prioritization Workgroup; 3) Feedback from Task Force members and other organisations; 4) Review by the Topic Prioritization Workgroup; 5) Final vote by the Task Force membership. A number of criteria for topics are considered. These include: What factors in current practice, or in the context of the culture, are important in understanding this clinical preventive service? Have major changes or controversies about this clinical preventive service emerged since the last recommendation was issued? If current practice issues seem important, consider whether background questions about current clinical practice need to be addressed here. |
| World Health Organization 2^nd^ edition [8] | 2014 | A six step approach as follows:  Step 1: generate an initial list of questions. The steering group develops an initial list of potential questions based on the agreed-upon scope of the guideline. These are then divided into background and foreground questions.  Step 2: draft the key questions in PICO format. The steering group, with input from the guideline development group, the systematic review team and the guideline methodologist, applies the PICO framework to the foreground questions.  Step 3: list relevant outcomes. The steering group should list relevant outcomes, including both the potential benefits and harms of the intervention or exposure. The guideline development group then reviews this list and may add additional outcomes considered important.  Step 4: review and revise. The list of questions and outcomes of interest should be sent by the steering group to the guideline development group and possibly to other stakeholders for review and comment.  Step 5: prioritize the key questions. The steering group, with input from the guideline development group, the systematic review team and the methodologist, finalizes the list of background and foreground questions and ranks them in order of priority. Determining which foreground questions need systematic reviews follows next.  Step 6: rate the outcomes. The guideline development group and/or other stakeholders, such as service users and others directly affected by the recommendations, then rate or rank the listed outcomes using a formal process, such as that described above.  Step 7: finalize the key questions and the important and critical outcomes. The steering group should list the high-priority key questions and the outcomes which the guideline development group will use to formulate recommendations. |
