# Supplementary material for: Defining key questions for clinical practice guidelines: a novel approach for developing clinically relevant questions
Source: Health Res Policy Syst. 2020 Sep 29;18:113. doi: 10.1186/s12961-020-00628-3 (PMC7523054; doi:10.1186/s12961-020-00628-3)
Supplement: Supplementary file 2 — Additional file 2. A summary of the steps and decisions involved in developing key clinical questions for the ‘Clinical guidelines for the diagnosis and management of work-related mental health conditions’. [file 12961_2020_628_MOESM2_ESM.docx]

**Additional File 2. A summary of the steps and decisions involved in developing key clinical questions for the ‘Clinical guidelines for the diagnosis and management of work-related mental health conditions’**

A summary of the steps and decisions made by the Guideline Development Group when developing key clinical questions is described below.

**STEP 1 Define the rationale**

A detailed needs analysis of patient outcomes, clinical practice, relevant policy and other research evidence was undertaken to explain the need for a guideline. This was presented to potential funding authorities when requesting funding to develop this guideline. At the first meeting of the Guideline Development Group, this rationale was further refined into a Scope document. Key elements of discussion included:

● Expanding determinants of health to include:

- Consequences of a mental illness diagnosis, such as inability to gain life insurance.
- The development of mental illness may be a slow process with no single overt precipitating incident.
- There is a difference between the development and the expression of a mental illness.
- The impact of a mental health condition can vary depending on circumstances.

● Terminology was discussed and a number of changes were made.

● The impact of personal issues on work-related mental health was added to the scope

● The inclusion and exclusion criteria were discussed and it was determined that an inclusive approach would initially be taken. All people engaged in work and all mental health conditions will be considered.

It was acknowledged that although the detailed management of comorbidities would not be considered it is important to remain cognisant of them

● It was suggested that the terminology in the Federal Government’s Mental Health Plan be reviewed and considered for adoption.

**STEP 2 Use qualitative research methods to determine the initial list of key questions based on the clinical challenges faced by target end-users**

The second step involves generating an initial list of questions that could be addressed in the guideline (Table 1). Step 2 involved:

(a) Identify key issues based on the problem areas or clinical dilemmas as identified in the qualitative research project.

(b) What questions will address these key issues?

(c) Map these questions to the Clinical Reasoning Framework

(d) Review existing guidelines and policies to identify current advice. A third literature search focussed on existing guidelines and systematic literature reviews. The results from this literature review were used to ascertain whether advice already exists to answer the clinical problems that were revealed during the qualitative research project.

(e) Generate initial list of questions.

First, a qualitative study was undertaken to reveal the clinical dilemmas that GPs face when caring for patients with work-related mental health problems. The findings from this study were organised into nodes and secondary nodes. Problem areas pertaining to each node were defined from the data. These problems were then transformed into questions that could be addressed in the guideline. Each question was then mapped onto the Clinical Reasoning Framework to highlight specifically when, during a clinical consultation, each problem was likely to arise. By referring to the Clinical Reasoning Framework here we are able to remind ourselves of the points during a consultation, which cause GPs the most difficulty.

Finally, we searched for existing advice that may influence the Guideline Development Group’s decision to include a question in a guideline. This includes the existence of guidelines or systematic literature reviews that potentially answer the guideline questions thus far. Following review of existing advice, the question for the guideline was either retained, revised, or a suggestion has been made to remove it altogether. Table 1 describes the steps taken to generate questions from the qualitative data.

*Table 1 STEP 2. Initial list of questions revealed in the qualitative study*

| **Node** | **Node- secondary category** | **Problem area** | **Question for guideline** | **Component of Clinical Reasoning Framework** | **Existing research or guidelines, position papers or SLRs that support or refute the need for this question**  ***(Australian guidelines highlighted)*** | **Content / message of existing guidelines, position papers, or SLRs** | **Revised question following Guideline Development Group discussion** | **Revised Component of the Clinical Reasoning Framework following Guideline Development Group discussion** |
| --- | --- | --- | --- | --- | --- | --- | --- | --- |
| Assessment | Disease specific | Knowing what screening tools are available for mental health and which are the best ones to use. Most GPs used the DSM-V criteria to diagnose depression. Not one GP mentioned ICD-10 codes (which include personality traits, lifestyle issues etc). Some use the K-10 some the DASS 21. Others noted that psych assessments & using structured assessments were important early on. One GP noted that they commonly prescribed SSRIs to patients experiencing panic attacks and SSNIs to patients that socially withdraw themselves. | Q1. Which tools should GPs use to diagnose stress/depression/anxiety (and differentiate between disorders)? | Diagnosis: Physical examination to get further data to confirm or refute the hypotheses | Dutch Guideline: The management of mental health problems of workers by occupational physicians; revised version, 2015 – *Not in English* | Two high quality Aus guidelines.  RANZCP guidelines are intended for psychologists, psychiatrists and physicians with an interest in mental health. | **Q1 revised. How to diagnose mood and stress disorders in general practice?** | No change. Keep component. |
|  |  |  |  |  | Canadian Taskforce: Recommendations on screening for depression in adults, 2013 |  |  |  |
|  |  |  |  |  | RANZCP: Clinical practice guidelines for mood disorders, 2015 |  |  |  |
|  |  | Problem with incorrect diagnoses: Once a diagnosis is made the issue has become medicalised. GPs are quick to diagnose when patient doesn’t meet diagnostic criteria which is problematic |  |  | USA: Adult depression in primary care, 2013 |  |  |  |
|  |  |  |  |  | Australian guidelines for the treatment of acute stress disorder & posttraumatic stress disorder, 2013 |  |  |  |
|  |  |  |  |  | Canada: DRAFT Medical Treatment Utilization Schedule. Mental Illness & Stress Guideline |  |  |  |
|  |  |  |  |  | BlackDogInst: DRAFT Diagnosis and Treatment of Post-traumatic Stress Disorder in Emergency Service Workers, 2016 |  |  |  |
| Assessment | Primary and secondary | It is important that GP considers other comorbidities that may contribute to depressive symptoms e.g. hyperthyroidism, anaemia, sleep disturbances, persistent pain, current medications, behavioural issues such as social isolation.  “I think sometimes GPs are very quick to diagnose things like adjustment disorder when the person doesn’t actually meet the diagnostic criteria and it’s very hard to then get that out of the person’s mentality.”CS2 | Q2. What comorbidities may contribute to depressive symptoms in patients with work-related mental injury? | Diagnosis: Could this patient have one of the masquerades in medical practice? | [APA: Practice guideline for the treatment of patients with major depressive disorder, third edition. 2015](https://www.guideline.gov/summaries/summary/24158/practice-guideline-for-the-treatment-of-patients-with-major-depressive-disorder-third-edition) | Comorbidities that are likely to occur as a result of work are not addressed in existing guidelines. | No change. Keep question. | No change. Keep component. |
|  |  |  |  |  | NICE: Depression in adults with a chronic physical health problem: recognition and management, 2015 |  |  |  |
|  |  |  |  |  | **A**ustralian guidelines for the treatment of acute stress disorder & posttraumatic stress disorder, 2013 |  |  |  |
| Assessment | Primary and secondary | Determining whether a mental health condition is work-related or not (resolution: understanding drivers and the causations and reasons behind it in order to address underlying problems. | Q3. How should a GP determine if a mental health condition has emerged as a result of work? What factors should be considered? What sources of information should be used? | Diagnosis:  Thoughtful and critical selection of investigations | SafeWorkAus: Dealing with workplace bullying-a workers guide | Defines bullying  Health services response  Systematic literature review is quite out of date. | No change. Keep question. | No change. Keep  component. |
|  |  |  |  |  | Dealing with workplace bullying-preventing and responding to workplace bullying |  |  |  |
|  |  |  |  |  | Systematic literature review: Psychosocial factors at work and risk of depression: a systematic review of the epidemiological evidence. 2008 |  |  |  |
|  |  |  |  |  | Systematic literature review: Work and depression/anxiety conditions: A systematic review of reviews. 2012 |  |  |  |
| Assessment | Primary and secondary | Being aware and checking for flags, particularly at certain timeframes (e.g. patient has been off work for more than 8 weeks after incident, ongoing workplace issues, or depressive signs following a traumatic event e.g. vehicle accident. | Q4. What flags indicate the emergence of a primary work-related mental health condition (for each common mental health diagnosis)? | Diagnosis: What is the probability diagnosis? Bullying as a masquerade. | NICE: Depression in adults with a chronic physical health problem: recognition and management, 2015 |  | Maybe combine with Question 1? |  |
|  |  |  |  |  | SLR: Prognostic factors for return to work in individuals with a common mental illness NOT YET PUBLISHED |  |  |  |
| Assessment | Primary and secondary | GPs over medicalising/misdiagnosing normal distress (suggested resolutions: use diagnostic criteria, DSM criteria (for diagnosing depression, anxiety), clinical framework, impact evidence, many GPs suggested the importance of referring early to a psychiatrist to make a diagnosis if they were unsure. | Q5. What are the optimal management strategies for "work-related stress"? (Can we draw on Acute-Stress guidelines?) | Management: Develop a management plan (a-d) | [**Australian guidelines for the treatment of acute stress disorder & posttraumatic stress disorder.**](https://www.guideline.gov/summaries/summary/49219/australian-guidelines-for-the-treatment-of-acute-stress-disorder---posttraumatic-stress-disorder) **2013** | Includes work-related. | No change. Keep question. | No change. Keep component. |
| Assessment | Non-improvement | When a patient's mental health does not improve e.g. ongoing workplace issues, non-improvement. | Q6. What flags indicate poor recovery following a diagnosis of a work-related mental health condition? | Diagnosis: Thorough by directed clinical history, with initial hypothesis generation and subsequent testing (a-e) | NICE: Depression in adults with a chronic physical health problem: recognition and management, 2015 |  | No change. Keep question. | No change. Keep component. |
|  |  |  |  |  | [Exploring interventions designed to support midwives in work-related psychological distress: a systematic literature review](https://www.nursingtimes.net/roles/mental-health-nurses/addressing-psychological-distress-in-midwives/7002592.article) |  |  |  |
| Assessment | Non-improvement | When a patient's mental health does not improve e.g. ongoing workplace issues, non-improvement. | Q7. What steps should a GP take to review a patient whose mental health is not improving, following a work-related mental health problem | Diagnosis: Thorough by directed clinical history, with initial hypothesis generation and subsequent testing (a-e) | NICE: Depression in adults with a chronic physical health problem: recognition and management, 2015 |  | No change. Keep question. | No change. Keep component. |
|  |  |  |  |  | SLR: Prognostic factors for return to work in individuals with a common mental illness NOT YET PUBLISHED |  |  |  |
| Assessment | Secondary | Being aware and checking for flags, particularly at certain timeframes (e.g. patient has been off work for more than a specified length of time after a musculoskeletal injury, ongoing workplace issues, asking for repeat scripts of opioid). GP being aware that the longer the patient is away from work (where they have a physical injury) the more likely they are to develop a secondary mental illness and not want to RTW. | Q8. What flags indicate the emergence of a mental health condition following an initial musculoskeletal injury? | Diagnosis: What is the probability diagnosis? | NICE: Depression in adults with a chronic physical health problem: recognition and management, 2015 | Does not include work-related injury specifically. | No change. Keep question. | No change. Keep component. |
| Assessment | Secondary | GPs anticipating that secondary mental health issues are likely to arise in patients with physical injuries. |  |  | NICE: [Improvement in the detection of depression and anxiety in long term sickness absence cases and the development of a care pathway](https://www.nice.org.uk/sharedlearning/improvement-in-the-detection-of-depression-and-anxiety-in-long-term-sickness-absence-cases-and-the-development-of-a-care-pathway), 2011 |  |  |  |
| Communication | Primary and secondary | A common issue that most GPs mentioned was lack of communication between b/w GP, employer, insurer and patient (together) (Resolution: GPs participating in case conferences early on) | Q9. Do case-conferences between health professionals improve the recovery outcome for patients who are diagnosed with a work-related mental health condition? What case-conferencing models are effective at improving patient recovery outcomes in a return to work context? | Diagnosis: Thoughtful and critical selection of investigations & Management: Develop a management plan for the presenting problem | [APA: Practice guideline for the treatment of patients with major depressive disorder, third edition. 2015](https://www.guideline.gov/summaries/summary/24158/practice-guideline-for-the-treatment-of-patients-with-major-depressive-disorder-third-edition) | RANZCP guidelines are high quality and include broad coverage of the topic. | Necessary? |  |
|  |  |  |  |  | RANZCP: Best practice referral, communication and shard care arrangements between psychiatrists, GPs and psychologists, 2014 |  |  |  |
| Communication | Throughout care | Often communication barriers between GPs and insurer (resolution: GP being proactive writing to insurer welcome communication, welcome call, provide an update. | Q10. How can a GP ensure that they receive useful feedback from clinicians and compensation agencies who are involved in a patient's care? | Management - procedural | RANZCP: Best practice referral, communication and shard care arrangements between psychiatrists, GPs and psychologists, 2014 | RANZCP guidelines are high quality and include broad coverage of the topic. | Necessary? |  |
| Communication | Assessment, management, non-improvement | Inconsistent advice being provided to patient as a result of health professionals not communicating amongst each other (Resolution: GP write/call treating physio/psych and get advice re. certification, discuss patient etc.) |  |  |  |  |  |  |
| Communication | Primary and secondary | A few GPs noted that lack of engagement with workplace was an issue due to confidentiality / patient consent (suggested resolution: seeking patients consent, having an occupational rehabilitation provider mediate between patient and workplace and communicate back to GP in certain jurisdictions) | Q11. When is it appropriate for a GP to contact a patient's workplace? How should the GP do this? | Management - procedural | None. |  | No change. Keep question. | No change. Keep component. |
| Communication | Management | Labels. Being conscious of language used on certificates. Considering how a diagnosis may effect patients mentality (e.g. putting on a cert that patient has depression, patient may see themselves as too sick to work). Ensuring diagnoses are accurate. Some GPs suggested using a provisional diagnosis e.g. adjustment disorder on DIS cert. or a non-specific (which is factually incorrect) diagnosis e.g. psychological injury'. | Q12. What factors should a GP take into account when communicating a work-related mental health condition diagnoses to a patient? | Management: 1.a-d (communicating the diagnosis with the patient) & Management procedural activity | RACGP: Redbook Chapter 10 Psychosocial  None for work-related. | Consideration of stigma. | No change. Keep question. | No change. Keep component. |
| Communication | Management | Rural patients concerns re. stigma (people in town finding out about their illness, this limiting work options in future - if they put a claim in) |  |  |  |  |  |  |
| Referral | Diagnosis and Management | Many GPs and CS workers mentioned its difficult for GPs to know how quickly should other health professionals (e.g. psychiatrists, psychologist etc.) be involved in the patient's care/ assessment? (referral is good if GP is unsure whether mhc work-related, outside GP scope, importance of second opinion medicine, aid time constraints and gain a team approach early ). "Stress" is particularly tricky. | Q13. When should a GP refer a patient to a psychiatrist / psychologist for diagnosis? When should a GP refer a patient to a psychiatrist / psychologist for management? | Diagnosis: Thoughtful and critical selection of investigations & Management: Develop a management plan for the presenting problem | RANZCP: Best practice referral, communication and shared care arrangements between psychiatrists, GPs and psychologists, 2014 | RANZCP guidelines are high quality and include broad coverage of the topic. | Necessary? |  |
| Referral | Diagnosis and Management | Knowing when to refer to a psychologist vs. a psychiatrists (some GPs suggested referring patient to psychiatrist for diagnosis) |  |  |  |  |  |  |
| Referral | Diagnosis and Management | Importance of early referral when there are flags for a psychological condition (especially in rural towns where wait times are long). Another GP worried that there were no psychologists in the area? In contrast, 1 GP noted it is important to not refer too early due to patient's mentality e.g. going to a psychologist I must be sick. |  |  |  |  |  |  |
| Management | Primary and secondary | Management of Stress is considered to be particularly tricky. | Q14. What are the treatment/management options for a patient with work-related stress? What are the recovery expectations of these strategies? When should a GP refer a patient to a psychiatrist / psychologist for management? | Management: Develop management plan (a-d) Management: Arrange follow up | WHO: WHO guidelines on conditions specifically related to stress, 2013 | Older or draft guidelines exist. | Remove question.  Q is similar to q5 and Q13. Remove.  Note, Q5 emerged from a concern to avoid miss-diagnosis of healthy stress as unhealthy stress. |  |
|  |  |  |  |  | Canada: DRAFT Medical Treatment Utilization Schedule. Mental Illness & Stress Guideline |  |  |  |
|  |  |  |  |  | SLR: Evidence-based Psychological Interventions in the Treatment of Mental Disorders: A Literature Review 2010 |  |  |  |
|  |  |  |  |  | SLR: A systematic review of psychological return-to-work interventions for people with mental health problems and/or physical injuries, 2006 |  |  |  |
|  |  |  |  |  | SLR: Improving return to work in adults suffering from symptoms of distress, 2012 |  |  |  |
|  |  |  |  |  | SLR: [Interventions to facilitate return to work in adults with adjustment disorders](http://onlinelibrary.wiley.com/doi/10.1002/14651858.CD006389.pub2/full), 2012 |  |  |  |
|  |  |  |  |  | SLR: Interventions to improve work outcomes in work-related PTSD: a systematic review, 2011 |  |  |  |
|  |  |  |  |  | SLR: [Interventions to improve return to work in depressed people](http://onlinelibrary.wiley.com/doi/10.1002/14651858.CD006237.pub3/full), 2014 |  |  |  |
|  |  |  |  |  | Web-based stress management for preventing stress and reducing sick leave in workers [Cochrane Protocol], NOT YET AVAILABLE Sep 2017 |  |  |  |
| Management | Primary and secondary | Influence of comorbidities: MSK problems, pain, opioid addiction, sleep disturbance, social isolation | Q15. Managing co-morbidities. | Throughout | Depression in adults with a chronic physical health problem: recognition and management (NICE 2015) https://www.nice.org.uk/guidance/cg91 | Good international guidelines. | Necessary? |  |
| Management | Non-improvement | When a patient does not improve after a primary work-related mental health problem e.g. ongoing workplace issues, non-improvement. | ~~Q16. What prevention strategies should be utilised for patients with a primary workplace-related mental health condition?~~ | Management: Develop management plan (a-d) | **WA.gov.au: Clinical guidelines for the physical care of mental health Consumers. 2010** |  | Delete. Same as questions 6 and 7. |  |
|  |  |  |  |  | **RACGP: Redbook Chapter 10 Psychosocial** |  |  |  |
|  |  |  |  |  | Canada: Best Practices for Return-to-Work/ Stay-at-Work Interventions for Workers with Mental Health Conditions 2010 |  |  |  |
| Patient perceptions, feelings & expectations | Management | Managing patient expectations was found to be challenging. E.g. if patient wants a prescription for opioids, complaining of pain, doesn’t want to RTW etc. Some GPs stated that they relied on the patient to explain what they wanted the GP to do, others said that they actively took on the role of decision-maker, thus guiding the patient about what to do. Some GPs said that they used to let their patients guide the trajectory but with experience they have learned that its better if the GP leads the way. (you’re never going to be 100% fit and that's okay. RTW is beneficial for your health etc. | Q17. What techniques are shown to improve the communication of unwanted information with patients? | Management: Develop management plan (a-d) |  |  | No change. Keep question. | No change. Keep component. |
| GP role | Claims | GPs feeling unsure / worried about the implications that their actions may have on a person's claim. Important GP doesn’t get into 'splitting' (e.g. siding with patient against workcover). Hear both perspectives (workplace and patient). GP advice to patient needs to be balanced and impartial (easy for GP to take on advocacy role and take 'patient's side' having only heard one side of the story. | Q18. What is the role of the general practitioner in the claims process of patients with work-related mental health conditions? | Management: Procedural |  |  | No change. Keep question. | No change. Keep component. |
| GP role | Claims | A number of GPs felt that they don't know enough about the claims process to be advising their patients about a claim. A number of GPs said that patients usually make up their mind about this themselves), others said they always tell a patient not to put in a claim because the process is too long, complex and stressful. |  |  |  |  |  |  |
| Options re. RTW | Management | GPs feeling concerned that they did not feel confident with determining the work options of a patient with MHC, especially if work has been the cause of the MHC. Does the GP's responsibility go beyond the capacity of the patient to include the capacity of the patient in their particular work situation? If so, how should they do this? (some GPs commented on the value of an Occupational Rehab Provider, some GPs advised their patients to find another job, some GPs - particularly for secondary MHC - found it useful to discuss options with the employer). | Q19. What factors should GPs consider to determine a person's capacity to (return to) work? What sources can GPs draw upon to determine a person's capacity to (return to) work? | Management: Develop management plan (a-d) | Canada: Best Practices for Return-to-Work/ Stay-at-Work Interventions for Workers with Mental Health Conditions 2010 | Canada: Best Practices for Return-to-Work/ Stay-at-Work Interventions for Workers with Mental Health Conditions 2010 | No change. Keep question. | No change. Keep component. |
|  |  |  |  |  |  | [HTA: Work-focused treatment of common mental disorders and return to work: a comparative outcome study, 2012](http://psycnet.apa.org/journals/ocp/17/2/220/) |  |  |
|  |  |  |  |  |  | ISCRR: Interventions to improve return to work outcomes in individuals with mental health conditions, 2015 |  |  |
| Options re. RTW | Management | Mediation: GPs encountered issues contacting the workplace (need to be diplomatic, patient's consent, finding time to do it etc, this is the occ rehabs role to undertake assessment, or RTW coordinator or psychologist, options may be discussed via case conferencing.) |  |  |  | [Interventions programs for enhancing return to work in individuals with a common mental illness, 2016](https://www.researchgate.net/profile/Yeshambel_Nigatu/publication/307968311_Interventions_for_enhancing_return_to_work_in_individuals_with_a_common_mental_illness_systematic_review_and_meta-) |  |  |
|  |  |  |  |  |  | [Exploring interventions designed to support midwives in work-related psychological distress: a systematic literature review](https://www.nursingtimes.net/roles/mental-health-nurses/addressing-psychological-distress-in-midwives/7002592.article) |  |  |
|  |  |  |  |  |  | RACP: [Health Benefits of Good Work Evidence Update](https://www.racp.edu.au/advocacy/division-faculty-and-chapter-priorities/faculty-of-occupational-environmental-medicine/health-benefits-of-good-work), 2013 |  |  |
| Options re. RTW | Management | Determining duties (occ rehab/ psych role to provide recommendations or surgeon if physical injury). GP may also chat to patient to better understand role and consider what they may or may not be able to do. Where it is a physical injury doing functional tests. |  |  |  | SLR: [Interventions for enhancing return to work in individuals with a common mental illness: systematic review and meta-analysis of randomized controlled trials 2014](https://www.ncbi.nlm.nih.gov/pubmed/27609709) |  |  |
|  |  |  |  |  |  | SLR: [Return to work coordination programmes for improving return to work in workers on sick leave](http://www.cochrane.org/CD011618/return-work-coordination-programmes-improving-return-work-workers-sick-leave), IN PROGRESS |  |  |
|  |  |  |  |  |  | SLR: [Theoretical frameworks for return to work in individuals with a common mental illness, IN PROGRESS](http://www.crd.york.ac.uk/PROSPERO/display_record.asp?ID=CRD42016033230) |  |  |
|  |  |  |  |  |  | SLR: [Return-to-work (RTW) interventions for employees with mental disorders: an overview of systematic reviews, IN PROGRESS](http://www.crd.york.ac.uk/PROSPERO/display_record.asp?ID=CRD42015023496) |  |  |
| Issues NOT raised but may be important | Management | Explore other preventive opportunities | Q20. Are work-related mental health conditions or WR MSK risk factors for addictive behaviours e.g. illicit drug and / or alcohol use? | Management - Explore other preventative opportunities |  |  |  |  |
| Issues NOT raised but may be important | Management | Accurate recording of diagnoses and management plans | Q21. Why should GPs record all patient diagnoses, investigations and management plans? | Management - procedural |  |  |  |  |

**STEPS 3 to 5: Convert the initial list of questions, Specify all relevant outcomes for each possible question, Review and revise draft key questions**

In order for the Guideline Development Group to select the key clinical questions that will be addressed in the guideline, it must first review and rate the possible outcomes in order of importance. Steps 3-5 of the Scope Development protocol outline a process of rephrasing the “Questions for guideline” into “systematic review questions” as follows:

Step 3 involves converting the initial list of questions, which were developed during step 2, into a Population Intervention Comparator Outcome format (Table 2).

Step 4 of the scope development process involves specifying all relevant outcomes for each possible questions. This includes not only those specified in the PICO but other desirable and undesirable outcomes. The final column titled “Secondary outcomes” has been left blank to allow the Guideline Development Group to populate this column during its discussion.

Step 5 of the scope development process involves rating the outcomes in order of importance for clinical decision-making.

The following changes were made, and the revised draft systematic review questions are included in Table 2.

**General**

- Question 17: “Unwanted information” was amended to “difficult information”.
- Question 20: The question was clarified to include opioids and gambling.

Question 1

- The question was re-written as “What tools can assist a GP in making a diagnosis of a work-related mental health condition”
- It was resolved that DSM-5 would be used to inform the terminology and that this would be made clear in the guideline.
- “Mood and stress disorders” was reworded as “mood, anxiety, stress and trauma related, and substance misuse disorders”, which are the conditions to be investigated.
- The population was discussed, particularly age and working status. The population was limited to workers. It was anticipated that the available evidence will be inherently biased to exclude adolescents.
- The clinical history as the basis of diagnosis and the use of diagnostic tools as adjuncts was emphasised. The question for the guideline was reworded to “What tools or instruments can assist in making a diagnosis?”.
- “Diagnostic methods” was clarified to mean “diagnostic instruments” or “diagnostic tools”.
- The distinction between assessment, screening and diagnostic tools was noted.
- Secondary outcomes were listed: overreliance on screening tools to make a diagnosis, over- and under notification and the effect on CALD patients.
- “DASS-24” was corrected to “DASS-21”.
- “DSM-V” was corrected to “DSM-5”.

Question 2

- The systematic review and guideline question was amended to refer to “psychiatric symptoms”
- The population was amended to refer to workers.
- *“Accurate diagnosis (non-mental health) Assessment tools (guidance on which to use)” was replaced with “Not missing an alternative diagnosis”*
- “In patients who attend general practice with symptoms of mental health conditions, what non-mental health conditions should be excluded before making a diagnosis of a mental health condition?” was replaced with “In patients who attend general practice with symptoms of psychiatric conditions, what non-psychiatric conditions should be excluded before making a diagnosis of a psychiatric condition?”
- The outcome was amended to refer to exclusion of an organic cause.
- The prospect of a long list of differential diagnoses was discussed.
- The secondary outcomes of economic burden and missing a psychiatric disorder were added.

Question 3

- Methods for assessment of work relatedness were discussed and ‘Corroborating information’ was added to the Intervention.
- Secondary outcomes added: In cases where compensation is possible, an assessment of work-relatedness can help to decide whether to pursue a claim.

Question 4

- “Accurate diagnosis” was removed from the outcomes.
- Secondary outcomes added: Better detection, Appropriate intervention, Work burden that might arise, Earlier referral pathways

Question 5

- “All treatment options” was replaced with “All management options”
- Secondary outcomes were added: system improvement to facilitate personal recovery, prevention of progression into psychiatric condition, stronger therapeutic relationship

Question 6

- Risk factors for slow improvement were discussed, including poor work performance, low attendance and suicide.
- Additional primary outcomes were added: Stigma, suicide attempts (or actual)
- Secondary outcomes were added: Flags to involve support from carers, wrong treatment, time for referral

Question 7

- The intervention was discussed and it was determined that “assessment strategies” be changed to “progress review”.
- “Return to work” was added to the primary outcome.
- Secondary outcomes were revised to: Reduce ‘stuck cases’, prevent early deterioration, prevent chronicity, stronger relationships between GPs and case managers, prevention of other problems.

Question 8

- The question was revised to “What are the predictors of a mental health condition following an initial musculoskeletal injury?
- Early anticipation of mental health problems was determined to be an outcome.
- Prevention of mental health problems and early intervention was listed as a secondary outcome.

Question 9 and 10

- Recent RANZCP guidelines were considered to be adequate.
- It was resolved that questions 9 and 10 would be removed.

Question 11

- Secondary outcomes were added: Feasibility of GP time, motivation, confidentiality, conflicting information from claims manager

Question 12

- The wording of the outcome was changed from “accurate diagnosis” to “accurate understanding of the diagnosis”.
- Secondary outcomes were added: All patient outcomes, GPs strategic approach to treatment, stigma, effect on GP/ patient relationship.

Question 13

- Question was removed as recently updated RANZCP guidelines already exist.

Question 14

- The question was considered vague and as such the comorbidities were defined as “substance related and addictive disorders” with an emphasis on gambling.
- It was noted that the PTSD guidelines had touched on the issue of comorbidities.
- The outcomes were listed as “evidence based management, improved recovery and return to work”.
- Secondary outcomes were listed as: confidentiality, reducing impact of comorbidities, patient outcomes and families

Question 15

- The question was removed as it was similar to question 12.

Question 16

- The question was removed as it sits outside the scope of the guideline
- It was considered that the question could be addressed in the introduction to the guideline.

Question 17

- Outcomes were discussed and several were added, namely: adopting a strengths based approach, vocational assessments, symptom and functional assessment (improving sleep, energy, activities and wider social engagement).
- Secondary outcomes were listed, namely: avoiding premature return to work and building patient resilience.

***Table 2 Outcomes from STEPS 3, 4 and 5 in the development of clinical questions***

| **Question for guideline** | **Population** | **Intervention** | **Comparator** | **Outcome** | ***Health setting*** | ***Systematic review question*** | **Secondary outcomes (step 4)** |
| --- | --- | --- | --- | --- | --- | --- | --- |
| Q1. What tools can assist a GP in making a diagnosis of a work-related mental health condition | Workers | Diagnostic methods | Existing diagnostic tools e.g., K-10, DASS-21, MMMSE, ICD-10 codes, DSM-5 criteria etc. | Accurate diagnosis of mental health condition | All. Consider primary care when crafting recommendation to assess applicability to general practice | Q1. In workers presenting with symptoms of mental health conditions, what diagnostic tools offer the most accurate (sensitive and specific) diagnosis of mood and / or stress disorders? | Over and under identification of patients  Cultural considerations |
| Q2. What comorbidities may contribute to psychiatric symptoms in patients with work-related mental injury? | Workers | Diagnostic tools | Existing tools for a range of disorders with similar symptomatology | Not missing an alternative diagnosis | Primary care | Q2. In patients who attend general practice with symptoms of psychiatric conditions, what non-psychiatric conditions should be excluded before making a diagnosis of a psychiatric condition? | Economic burden  Missing a psychiatric disorder |
| Q3. How should a GP determine if a mental health condition has emerged as a result of work? What factors should be considered? What sources of information should be used? | Patients who attend primary care with a diagnosed mental health condition | Investigative methods,  Corroborating information | Existing scales and methods | Accurate assessment of the contribution of work to the MHC | Primary care  Secondary care | Q3. In patients with a diagnosed mental health condition, what methods are effective at indicating the probability that the diagnosed mental health condition has arisen as a result of work? | In cases where compensation is possible, this helps to decide whether to pursue a claim.  Confidentiality issues that might arise. |
| Q4. What flags indicate the emergence of a primary work-related mental health condition (for each common mental health diagnosis)? | Workers | Flags | Flags that indicate particular mental health conditions (e.g. PTSD, GAD, depression) | Early detection | Any setting. E.g. at work, at home, socially. | Q4. In workers, what flags accurately facilitate the early detection of a work-related mental health condition? | Better detection  Appropriate intervention  Work burden that might arise  Earlier referral pathways |
| Q5. What are the optimal management strategies for "work-related stress"? | Patients with work-related stress | Management options | All management options | Personal recovery  Return to work |  | Q5. In patients with work-related stress, what management strategies result in the highest levels of personal recovery?  Q6. In patients with work-related stress, what management strategies result in the highest rates of return to work? | System improvement to facilitate personal recovery  Prevention of progression into psychiatric condition  Stronger therapeutic relationship |
| Q6. What flags indicate poor recovery following a diagnosis of a work-related mental health condition? | Patients with work-related mental health conditions | Symptoms and signs that indicate poor recovery  e.g. risk factors about chronicity | Symptoms and signs that do not indicate slow recovery | Early detection of slow patient recovery  Patient satisfaction (re. recovery)  Stigma  Suicide attempts (or actual) | Work, social, primary care | Q7. In patients with a diagnosis of a work-related mental health condition, what symptoms and signs indicate slow patient recovery? | Over diagnosis  Narrowing the focus on work  Flags to involve support from carers  Wrong treatment, time for referral etc |
| Q7. What steps should a GP take to review a patient whose mental health is not improving, following a work-related mental health problem | Patients with work-related mental health conditions | Review (wholistic)  (include barriers) | Assessment upon initial presentation | Patient recovery  Patient satisfaction |  | Q8. In patients with work-related mental health conditions who are not meeting recovery expectations, what strategies should a general practitioner undertake to improve patient recovery?  (e.g. identify recurrent/continuing stressors, new symptoms/signs, new comorbidities such as D&A use) | Reduce ‘stuck cases’  Prevent early deterioration  Prevent chronicity  Stronger relationships between GPs and case managers  Prevention of other problems |
| Q8. What flags indicate the emergence of a mental health condition following an initial musculoskeletal injury? | Patients with work-related musculoskeletal injuries | Signs and symptoms of emerging mental health conditions  (e.g. enhanced pain response etc) | Sign and symptoms commonly associated with MSK injury (not mental health related) | early anticipation of secondary mental health condition |  | Q9. In patients with work-related musculoskeletal injuries, what signs and symptoms are indicative of an emerging mental health problem? | Prevention of chronicity  Early addressing of problems |
| ~~Q9. Do case-conferences between health professionals improve the recovery outcome for patients who are diagnosed with a work-related mental health condition?~~  ~~What case-conferencing models are effective at improving patient recovery outcomes in a return to work context?~~ | ~~Patients with work-related mental health conditions~~ | ~~Case conference~~ | ~~Case conference models~~ | ~~Patient recovery~~  ~~Return to work~~  ~~Improved communication~~ | ~~Primary care~~  ~~Secondary care~~ | ~~Q10. In patients diagnosed with a work-related mental condition, which case conference models improve patient recovery outcomes?~~  ~~Q11. In patients with work-related mental health conditions, which case conference models are effective at improving patient return to work?~~ |  |
| **~~Q10.~~** ~~How can a GP ensure that they receive useful feedback from clinicians and compensation agencies who are involved in a patient's care?~~ | ~~GPs~~  ~~Clinicians~~  ~~Compensation agencies~~ | ~~methods of communication~~ | ~~Various communication methods~~ | ~~prompt and useful feedback about a patient~~  ~~Improved communication~~  ~~Patient satisfaction~~ | ~~Primary care~~  ~~Secondary care~~ | ~~Q12. For GPs who are managing patients with compensation claims, what communication methods are effective at ensuring the receipt of prompt and accurate feedback regarding a patient’s care?~~ |  |
| Q11. When is it appropriate for a GP to contact a patient's workplace? How should the GP do this? | GPs with patients who describe work-related mental health problems | appropriate communication with the workplace | GPs not contacting the workplace | Appropriate investigation of a work-related mental health condition.  Avoidance of issue with employer.  Patient satisfaction  Patient may RTW early (if mediation occurs early) | Primary care  Work | Q13. For GPs who are managing patients with compensation claims, what is appropriate communication with the patient’s workplace, in order to appropriately investigate a work-related mental health condition? |  |
| Q12. What factors should a GP take into account when communicating a work-related mental health condition diagnoses to a patient? | Patients diagnosed with a work-related mental health condition | Factors that must be considered prior to communicating a diagnosis | Non-specific factors | Patient outcome(s) to be defined….e.g. patient acknowledgement and understanding of the diagnosis, avoidance of unnecessary stress.  Clear communication  Accurate understanding of the diagnosis  Management of patient expectations re. recovery | Primary care | Q14. When conveying a diagnosis of a work-related mental health condition to a patient, what factors should GPs consider, to ensure that their diagnosis is understood and acknowledged by the patient? | All patient outcomes but for employers  GPs strategic approach to treatment  Stigma  Effect on GP/ patient relationship |
| ~~Q13. When should a GP refer a patient to a psychiatrist or psychologist for diagnosis? When should a GP refer a patient to a psychiatrist or psychologist for management?~~ | ~~Patients with work-related mental health concerns~~ | ~~Time of referral~~  ~~Or~~  ~~Signs that indicate need for referral~~ | ~~Referral (non- time specific)~~ | ~~Accurate diagnosis~~  ~~Early referral – RTW earlier, skew patient perceptions (e.g. labels – patient sick)~~  ~~Late referral – RTW later,~~  ~~Multidisciplinary coordination of care~~ | ~~Primary care~~  ~~Secondary care~~ | ~~Q15. In patients with work-related mental health concerns, what signs indicate the need for referral to a psychiatrists or psychologist for an accurate diagnosis/ management?~~ |  |
| Q14. Managing mental health co-morbidities. | Patients with co-morbidities | Interventions to manage mental health comorbidities  (specifically related and addictive disorders) | Compare between interventions | Evidence-based management of co-morbidities | Primary care | Q16. In patients with co-morbidities, what interventions are effective at improving the provision of evidence-based management of comorbidities by GPs? | NB: Look at PTSD guidelines  Confidentiality  Reducing impact of comorbidities  Patient outcomes and families |
| ~~Q15. What techniques are shown to improve the communication of unwanted information with patients?~~ | ~~Patients~~ | ~~Communication techniques~~ | ~~No communication techniques being used~~ | ~~Patient outcome(s) to be defined….e.g. patient acknowledgement and understanding of the diagnosis, avoidance of unnecessary stress.~~ | ~~Primary care~~  ~~Secondary care~~ | ~~Q17. In general practice patients, what techniques can be used by a GP to improve the communication of unwanted information to their patients?~~ |  |
| ~~Q16. What is the role of the general practitioner in the claims process of patients with work-related mental health conditions?~~ | ~~General practitioners with patients who apply for a claim~~ | ~~Educational or systemic~~ |  | ~~GP confidence in their capacity to perform the role~~ | ~~Primary care~~ | *~~Not a question for a guideline. Include in discussion.~~* |  |
| Q17. What factors should GPs consider to determine a person's capacity to (return to) work? What sources can GPs draw upon to determine a person's capacity to (return to) work? | Workers | Factors that determine a person’s capacity to (return to) work  (eg recovery goal setting) | All factors regarding patient care | RTW / personal recovery  Managing patient expectations  Patient satisfaction  Adopting or utilising a strength-based approach  Vocational assessments  Improvement of sleep disorders, function, social interaction | Primary care  Work | Q17. In workers, what information should a GP consider to determine whether a person is capable and has capacity to (return to) work? | Prevent inappropriate and premature return to work  Build resilience |

**STEP 6 and 7: Rate the outcomes in order of importance for clinical decision-making**

Guideline Development Group members first rated the primary outcome for each question. Then individual ratings were collated by a non-voting member of the team and presented back to the panel. The panel discussed differences in ratings and through consensus agreed on a rating for the primary outcome of each question. The consensus-developed ratings are presented in Table 3. Together, the panel then ranked the questions in order of importance to address in the guideline (Table 3).

***Table 3. Rating of outcomes and ranking of draft questions for the clinical guideline***

| **Ranking**  **(In order of agreed importance)** | **Final version of systematic review question considered and scored** | **Primary outcome** | **Primary outcome rating score (1 = not important to 5 = very important)** | **Discussion** | **Further changes made from draft version (table 2)** |
| --- | --- | --- | --- | --- | --- |
| Q1 | In workers presenting with symptoms of mental health conditions, what diagnostic tools offer the most accurate (sensitive and specific) diagnosis and determination of severity of mood, anxiety, stress and trauma related, and substance misuse disorders. | Accurate diagnosis of mental health condition | 5 | The importance of determining severity. | Determination of severity included in the final question. |
| Q2 | In patients who attend general practice with symptoms of psychiatric conditions, what non-psychiatric conditions should be excluded e.g. thyroid disease. | Not missing an alternative diagnosis | 1 | GPs are trained to perform this task. | Question removed |
| Q3 | In patients with a diagnosed mental health condition, what methods are effective at indicating the probability that the diagnosed mental health condition has arisen as a result of work? | Accurate assessment of the contribution of work to the mental health condition | 5 |  | The word “tools” was changed to “methods”. |
| Q4 | In workers, what factors assist in the early detection of a work-related mental health condition? | Early detection | 4 | This question overlaps with | Replaced “flags that accurately facilitate” with “factors that assist in the”. |
| Q5 | In patients with work-related stress, what strategies result in the highest levels of personal recovery? | Personal recovery | 5 |  | Replaced “management strategies” with “strategies”. |
| Q6 | In patients with work-related stress, what GP strategies result in the highest rates of return to work? | Return to work | 5 | Q5 and Q6 can be combined | Replaced “management strategies” with “GP strategies”. |
| Q7 | In patients with a diagnosis of a work-related mental health condition, what signs and symptoms would lead to delayed progress in the patient’s condition? | Early detection of slow patient recovery | 5 | “Features, factors, signs and symptoms” are to be built into the search strategy. Outcomes could include suicidal ideation and attempts. | Replaced “recovery” with “progress”.  Replaced “symptoms and signs” with “signs and symptoms”.  Replaced “indicate” with “would lead to”.  Replaced “poor patient progress” to “delayed progress in the patient’s condition”. |
| Q8 | In patients with work-related mental health conditions who are not improving, what strategies should a general practitioner undertake to improve the patient’s condition? | Patient recovery | 5 |  | Replaced “meeting recovery expectations” with “improving”.  Replaced “improve patient recovery” with “improve the patient’s condition”. |
| Q9 | In patients with work-related musculoskeletal injuries, what signs and symptoms are indicative of an emerging mental health problem? | Early anticipation of secondary mental health condition | 1 | There is similarity with question 4.  There is concern the question is moving out of scope. | Question removed. |
| Q13 | For GPs who are managing patients with compensation claims, what is appropriate communication with the patient’s workplace, in order to appropriately investigate and manage a work-related mental health condition? | Appropriate investigation of a work-related mental health condition. | 5 | The role of case manager and GP.  The importance of communication. | Replaced “investigate” with “investigate and manage”. |
| Q14 | When conveying a diagnosis of a work-related mental health condition to a patient, what factors should GPs consider, to ensure that their diagnosis is understood and acknowledged by the patient? | Patient acknowledgement and understanding of the diagnosis | 5 |  |  |
| Q16 | In patients with work related mental health conditions what interventions are effective at managing comorbid substance related and addictive disorders. | Evidence-based management of co-morbidities | 4 | The challenges associated with managing comorbid conditions were discussed. | Amended the systematic review question to “In patients with work related mental health conditions what interventions are effective at managing comorbid substance related and addictive disorders.” |
| Q17 | In workers, what information should a GP consider to determine a person’s capacity to (return to) work? | Personal recovery, RTW  Managing patient expectations  Patient satisfaction  Adopting or utilising a strength-based approach  Vocational assessments  Improvement of sleep disorders, function, social interaction | 5 | Is this a part of GP management? |  |

The Guideline Development Group agreed on the following list of questions (Table 4):

***Table 4. Final list of clinical questions***

|  | POPULATION | INTERVENTION | COMPARATOR | OUTCOME |
| --- | --- | --- | --- | --- |
| 1. In workers presenting with symptoms of mental health conditions what tools can assist a GP to make an accurate (sensitive and specific) diagnosis of a mental health disorder and its severity? | Patients who are in active employment | Diagnostic methods | Existing diagnostic tools e.g., K-10, DASS-21, MMMSE, ICD-10 codes, DSM-5 criteria etc. | Accurate diagnosis of mental health condition |
| 2. In workers what factors assist in the early detection of a comorbid work-related mental health condition? | Patients who are in active employment and have been diagnosed with a mental health condition | Flags that indicate particular mental health conditions (e.g. PTSD, GAD, depression) | Flags that indicate particular mental health conditions (e.g. PTSD, GAD, depression) | Early detection |
| 3. In patients with a diagnosed mental health condition what methods are effective at indicating the probability that the diagnosed mental health condition has arisen as a result of work? | Patients who have been diagnosed with a mental health condition (depression, anxiety, PTSD, acute stress disorder, adjustment disorder or substance use disorder) | Investigative methods,  Corroborating information | Existing scales and methods | Accurate assessment of the contribution of work to the mental health condition |
| 4. When conveying a diagnosis of a work-related mental health condition to a patient what factors should GPs consider to ensure that their diagnosis is understood and acknowledged by the patient? | Patients who are in active employment and who present with a mental health condition | Factors that must be considered prior to communicating a diagnosis | Non-specific factors | Patient acknowledgement and understanding of the diagnosis |
| 5. In patients with a work-related mental health condition what GP strategies result in the highest level of personal recovery and/or return to work? | Patients who are in active employment and have been diagnosed with a work-related mental health condition | Management options | All management options | Personal recovery / return to work |
| 6. In workers with a mental health condition what information should a GP consider to determine whether a person has capacity to work? | Patients who are in active employment and have been diagnosed with a work-related mental health condition |  |  | Personal recovery, RTW  Managing patient expectations  Patient satisfaction  Adopting or utilising a strength-based approach  Vocational assessments  Improvement of sleep disorders, function, social interaction |
| 7. What is appropriate communication with the patient’s workplace in order to appropriately manage a work-related mental health condition? | Employers | Appropriate communication with the workplace | GPs not contacting the workplace | Appropriate investigation of a work-related mental health condition. |
| 8. In patients with a work-related mental health condition what GP interventions are effective at managing comorbid substance misuse and addictive disorders? | Patients who are in active employment and have been diagnosed with a work-related mental health condition | Interventions to manage mental health comorbidities (specifically related and addictive disorders) | Compare between interventions | Evidence-based management of co-morbidities |
| 9. In patients with a diagnosis of a work-related mental health condition what factors adversely affect progress in the patient’s condition? | Patients who are in active employment and have been diagnosed with a work-related mental health condition | Symptoms and signs that indicate slow recovery e.g. risk factors about chronicity | Symptoms and signs that do not indicate slow recovery | Early detection of slow patient recovery |
| 10. In patients with work-related mental health conditions who are not improving what strategies should a GP undertake to improve the patient’s condition? | Patients who are in active employment and have been diagnosed with a work-related mental health condition | Management and assessment options | Management and assessment options | Patient recovery |
